# Research on the "shape-performance-control" integrated digital twin system for boom-type roadheaders

**DOI:** 10.1038/s41598-024-56539-8

**Published:** 2024-03-09

**Authors:** Jianzhuo Zhang, Chuanxu Wan, Jie Wang, Ce Chen, Tao Wang, Runfeng Zhang, Hao Guo

**Affiliations:** 1 College of Mechanical Engineering, Liaoning Technical University, Fuxin, 123000 China; 2 Tianjin Key Laboratory for Advanced Mechatronic System Design and Intelligent Control, School of Mechanical Engineering, Tianjin University of Technology, Tianjin, 300384 China

**Keywords:** Boom-type roadheader, Digital twin, Recurrent neural network, Shape-performance-control integration, Energy infrastructure, Mechanical engineering

## Abstract

The boom-type roadheader plays a crucial role in coal mining. However, conducting the real-time monitoring of the mechanical performance and comprehensive adaptive cutting in the dynamic cutting process are challenging. To address these issues, a digital twin system that integrates the elements of “shape, performance, and control” for roadheaders is presented in this paper. The system comprises three components: physical space, service space, and twin space. The service space forms the core of the entire system. Within this space, twin models and control models are created using numerical simulation, artificial intelligence and multi-source data fusion technology. These models serve the purpose of predicting the roadheader’s mechanical performance and controlling the swing speed of the cutting arm. The physical space is built using technologies such as robot kinematics, electrical systems, hydraulic transmission, and other relevant techniques. This approach facilitates the transmission of multi-sensor data to twin models. The control model then manages the roadheader’s function based on the output signals from the control model. The twin space is constructed utilizing physical rendering engines, databases, and 3D modelling tools. This space visualizes and stores the movement, performance, and control parameters of the roadheader. The results demonstrate that the average absolute error between the measured data from the test’s three position strain gauges and the predicted data from the twin system is 10.38 MPa. Furthermore, the twin system achieves an average update interval of 0.34 s, allowing real-time stress monitoring of the structural components of the roadheader and preventing damage caused by overload. The proposed control model enables adaptive adjustment of the swing speed of the cutting arm in approximately 0.3 s. This improvement significantly enhances the adaptive cutting capabilities of roadheaders when dealing with complex coal and rock formations.

## Introduction

With the continuous progress of digitalization, the Internet of Things, and artificial intelligence technology, the coal industry has continued to promote intelligent transformation and development. The boom-type roadheader is an indispensable piece of equipment for coal mining. However, the environment of the comprehensive digging face is dangerous. Workers often operate the roadheader in an environment with high temperature and humidity, significant amounts of dust and poor visibility; and are unable to intuitively, comprehensively and timely understand the working state of the roadheader, resulting in the roadheader being in a "blind operation" state. Moreover, due to the complexity and diversity of coal and rock occurrence conditions, the density and hardness of the coal and rock cut by roadheaders are constantly changing^1–3^, resulting in large fluctuations in the output power of the cutting motor and the stress state of the structural parts, and overload and underload often occur^4–6^. Therefore, the digitization of the roadheader shape, mechanical performance parameters, and intelligent control are the key technologies for reducing the failure rate of roadheaders, improving the efficiency of roadheaders, and ensuring worker safety.

Digital twin technology bridges physical space and digital space mapping and can be used to digitize natural objects or processes in the physical world into virtual models to achieve accurate simulation, optimal design, and intelligent control of physical world objects. Based on the twin model proposed by Grieves^8^, which consists of a physical entity, twin entity, and data interaction, Tao et al.^7^ added two dimensions of twin data and service to construct a five-dimensional digital twin model. They verified the accuracy of the five-dimensional digital twin model with the case of a wind turbine digital twin. To determine the geometric shape and mechanical performance of significant equipment, Song et al.^9^ proposed a multi-fidelity prediction model of "calculation and measurement fusion" and "shape-performance integration," which was jointly driven by a mechanism model and sensing data to realize relatively high-precision approximate real-time monitoring of significant equipment. Additionally, they built a mapping model integrating an analytical model, numerical model, and artificial intelligence model. By completing the integrated monitoring of boom crane shape performance^10^, He et al.^11^ developed a multi-level fusion modelling method that combined the advantages of different agent models and used simulation and measurement data to improve the stress prediction accuracy of critical parts of large complex mechanical structures.

Several studies have focused on monitoring the conditions of roadheaders. As an advanced digital technology, digital twin technology has also been widely applied in fields such as coal and tunnel excavation. Latif et al.^12^ used machine learning, visualization and monitoring to predict the performance of TBMs and complete the prediction, visualization and monitoring of tunnel construction progress. Zhang et al.^13^ proposed a digital twin-driven roadheader virtual teaching memory cutting control method. By utilizing the characteristics of digital twin technology and virtual reality technology, a virtual teaching strategy under complex working conditions was studied, providing ideas for memory cutting and intelligent control of roadheaders. Cai et al.^14^ achieved functions such as mining process simulation, data augmentation visualization, remote control, and intelligent fault detection by integrating technologies such as the Internet of Things, the internet, digital twins, and augmented reality. Lv et al.^15^ designed a structured communication mechanism for transmitting real-time excavation data around a physical data-driven TBM virtual excavation simulation and proposed a data-driven TBM virtual excavation simulation method based on Unity3D. Ding et al.^16^ proposed a shearer health state prediction method driven by digital twins and deep learning and built a shearer digital twin to visualize the shearer's working state and health state prediction. A prediction model of the remaining life of critical parts of the shearer based on deep learning was established to improve the prediction and management of the shearer's health status. Wang et al.^17^ analysed the static characteristics, dynamic characteristics, behaviour rules, and interaction relationships of heterogeneous elements of excavation faces given the requirements of virtual and real interactive control of excavation scenes. They built an intelligent digital twin model of the excavation face to accurately describe the excavation workflow. Liu et al.^18^ visualized the working state of a roadheader. They predicted faults by using information such as images, temperature, pressure, and vibration. Moreover, they built a multi-information monitoring system for the roadheader's working environment, cutting state, oil state, and life prediction of critical components.

On the other hand, adaptive control during the cutting process is an urgent problem. Ma et al.^19^ analysed the research status of intelligent excavation at home and abroad and proposed a shape cutting control method based on vision technology and an adaptive cutting control method based on genetic algorithm optimization for intelligent cutting. Wang et al.^20^ took the current of the cutting motor, the vibration acceleration of the cutting arm and the pressure of the hydraulic cylinder as the judgement basis for load identification, used the improved BP neural network algorithm of particle swarm optimization to determine the cutting load, and completed the swing speed control of the cutting arm through the improved simulated annealing particle swarm optimization (ISAPSO) fuzzy PID controller. Thus, the adaptive control of the roadheader can be realized. Wang et al.^21^ used the identification method of a multi-neural network and evidence theory combined with multi-sensing information to determine the dynamic load of a roadheader and improved the confidence of roadheader load identification.

These scholars have made important contributions to monitoring the working state of roadheaders and adaptive cutting. However, few scholars have conducted research on the real-time monitoring of the mechanical performance of roadheaders. Sensors such as acceleration sensors, pressure sensors, displacement sensors and cameras are still used to monitor the working state of roadheaders, and the mechanical performance of roadheaders cannot be obtained in real time. In addition, most of the existing adaptive cutting methods for roadheaders reflect the load state of the roadheader through signals such as cutting arm vibration acceleration, cutting motor current and hydraulic cylinder pressure^22^; subsequently, feedback control is performed on the cutting parameters. This method can only reflect the load state of local areas of roadheaders and cannot fully reflect the service performance of roadheaders.

Due to the lack of in-depth real-time monitoring of the mechanical performance of roadheaders and the incomplete basis of adaptive control, an integrated digital twin system of "shape-performance-control" of roadheaders is proposed for the real-time monitoring and evaluation of "shape-performance-control" information. The main contributions of this paper are as follows:

1. Numerical simulation models of roadheaders under different working conditions were established, and various deep learning models were used to predict the mechanical performance of roadheaders. The models' accuracy, rapidity, and stability were considered comprehensively, and the optimal models were selected. This study fills the gap in the real-time monitoring of the mechanical performance of roadheaders and provides a new dimension for the on-line monitoring of roadheaders.
2. The "shape-performance-control" integrated digital twin system of roadheaders was built to monitor the mechanical performance parameters of roadheaders in real time, effectively avoid the occurrence of overload conditions, and evaluate and warn of the service performance of roadheader structural parts.
3. By applying the control method of fusing a neural network and model predictive control (MPC), the swing speed of a roadheader was controlled by the extreme stress of the roadheader, which provides a new basis for intelligent roadheader control.

## "Shape-performance-control" integrated digital twin system

### Method

**Figure 1:**
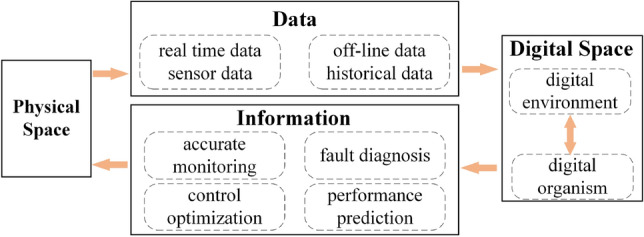
Digital twin structure flow.

To address the issues of real-time stress monitoring and adaptive control of roadheaders used in coal mines, an integrated method for constructing a digital twin system called "shape-performance-control" is proposed. The digital twin structure flow is shown in Fig. 1:

This method utilizes the digital twinning process to input physical information into the digital sphere, extract significant data, and conduct precise monitoring, fault detection, performance prediction, and control optimization of physical entities. By mapping the virtual and real relationships in digital twins, an integrated “shape-performance-control” digital twin system for roadheaders is constructed utilizing the following steps:

1. Finite element technology is utilized to conduct the numerical simulations of roadheaders, generating the necessary dataset for training the twin model.
2. A twin model is built using deep learning technology to predict roadheader stress.
3. A neural network-MPC model is constructed to regulate the swing speed of the cutting arm based on the stress predicted by the twin model.
4. Multi-sensor data are input to the twin model and control model to obtain the necessary data to construct the digital twin.
5. The digital twins are rendered and displayed in the physics engine to visualize the data accumulated by the twin model and control model.

The roadheader “shape-performance-control” integrated digital twin system offers several advantages, particularly as it introduces the concept of “shape-performance-control” for the first time in the realm of industrial and mining equipment. This system completes the closed-loop process from form monitoring to performance monitoring and feedback control, thereby preventing overload damage to the roadheader and increasing the intelligence level of the mining equipment.

### System structure and workflow

**Figure 2:**
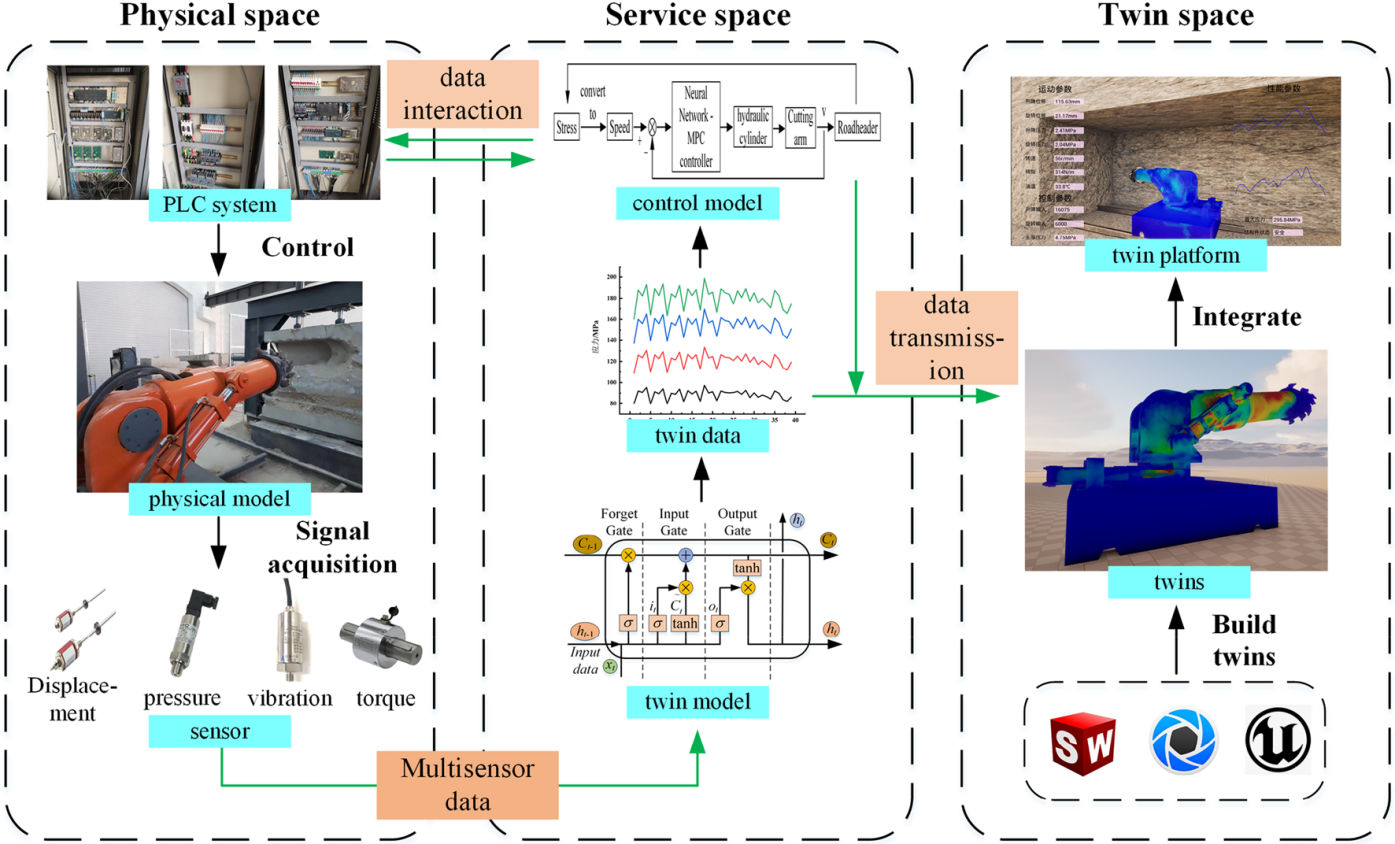
"Shape-performance-control" integrated digital twin system of a boom-type roadheader.

The "shape-performance-control" integrated digital twin system of roadheaders includes physical space, service space and twin space, and the system composition is shown in Fig. 2.

In Fig. 2, the physical space is the virtual-real mapping object of the digital twin. The multi-sensor data of the actual physical model are input into the service space. Subsequently, the twin model predicts the mechanical performance of the roadheader, and the control model carries out feedback control.

The service space is the core of the digital twin system of the “shape-performance-control” of the roadheader. The service space contains a twin model and a control model. The twin model is trained by the dataset generated by numerical simulation. Multi-sensor data are used to predict the mechanical performance of the roadheader. The control model makes closed-loop adjustments to the cutting parameters based on roadheader stress extremes.

The twin space embodies the integration of the "shape-performance-control" of the roadheader. The sensor data and twin data are used to generate the twin of the roadheader in virtual space, and the twin data and sensor data are integrated into the twin platform, which is convenient for observing changes in the roadheader state, evaluating the mechanical performance of the roadheader, and visualizing the roadheader "shape-performance-control" parameters in real-time.

## Development of a twin system

In Sect. "Method", the construction process of the integrated digital twin system for the “shape-performance-control” of the roadheader is described. Additionally, the specific implementation details are introduced in this section.

### Dataset construction

To construct the dataset for training the twin model of the roadheader, the mechanical performance of the machine, including the stress and deformation, is calculated through numerical simulation. The cutting resistance generated by the cutting head when cutting coal and rock is the main load on the machine. This cutting resistance can be measured using multiple sensors. The mechanical performance of the roadheader is represented by the following eight variables: the vibration acceleration of the cutting arm, displacement of the piston rod of the rotating hydraulic cylinder, displacement of the piston rod of the lifting hydraulic cylinder, pressure of the left rotating hydraulic cylinder, pressure of the right rotating hydraulic cylinder, pressure of the lifting hydraulic cylinder, speed of the cutting head and torque of the hydraulic motor. The LS-DYNA module of ANSYS is used to conduct parametric numerical simulation of the roadheader, which involved multiple steps. Here is a brief explanation of the important steps in the basic process of finite element analysis:

#### Model preprocessing

The three-dimensional model of the roadheader is imported into Spaceclaim. In this step, parts such as bolts and nuts that have little influence on the mechanical properties of the roadheader are deleted. Additionally, chamfering is removed, and the hydraulic cylinder, cutting arm, and other parts are merged separately to simplify the model.

#### Material setting

After the preprocessing, materials are assigned to each part of the model. The roadheader base is made of Q235 material, the rotating table is made of ZG35 material, the lifting and rotating hydraulic cylinder is filled with No. 46 anti-wear hydraulic oil, the cutting head is treated as a rigid body, and other parts are made of No. 45 steel. The parameters of each material are provided in Table 1.

**Table 1:** Parameters of the materials.

Material	Density/(kg/m^3^)	Young’s modulus/GPa	Poisson’s ratio	Yield strength/MPa
45steel	7830	209	0.274	355
Q235	7850	207	0.3	235
ZG35	7800	207	0.3	350
L-HM46	850	–	–	–

#### Meshing

The mesh type of hard structural parts are set to Tetrahedrons, and the liquid mesh type is set to Automatic. The cutting head is considered rigid and does not undergo plastic deformation, so its mesh is roughly divided. This means that the mesh is not as refined as other components. The rotating and lifting hydraulic cylinder is an important power component, and its mesh should be encrypted, and the mesh size was set to 15 mm. The contact surface of hydraulic cylinder, cutting arm and rotating table are encrypted with grid. The mesh size was set to 10 mm. For other parts, the mesh size was set to 30 mm. The total number of elements in the system is reported to be 278,747.

#### Contact setting

The friction contact is set between the piston of the lifting and rotating hydraulic cylinder and the inner wall of the cylinder. The friction coefficient for this contract is 0.15, and the frictionless contact is set between other components.

#### Boundary condition setting

The boundary condition is applied at the corresponding part position, and the set value of the boundary condition is determined based on the measurement from the sensor during the actual cutting process. Specifically, fixed constraints are imposed on the surface of the roadheader base, torque and speed are applied at the cutting head, and remote displacement is applied to the front end face of the cutting arm to simulate the vibration during the cutting process. It worth nothing that the cutting arm primarily vibrates in the plane parallel to the coal wall, with minimao vibration in the direction perpendicular to the coal wall. The specific values of the boundary conditions are provided in Table 2.

**Table 2:** The value range of the boundary conditions.

Speed range/(r/min)	Torque range/(N/m)	X displacement range/mm	Y displacement range/mm	Z displacement range/mm
0 ~ 105	0 ~ 4000	− 30 ~ 30	− 30 ~ 30	− 10 ~ 10

**Figure 3:**
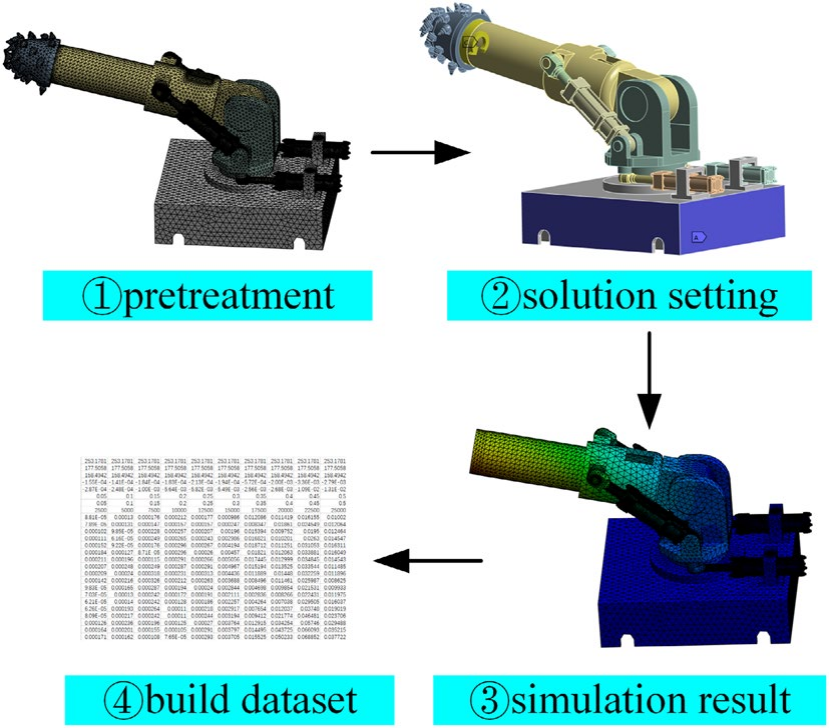
Dataset construction process.

The stress results of each node of the roadheader are integrated, resulting in the mechanical performance dataset of the roadheader, which is required for twin model training. The dataset construction process is shown in Fig. 3.

The correlation between the twin model and the variables and stresses in the dataset can be mathematically described by Eq. (1).

1 $$ \begin{gathered} F([x_{1} ,x_{2} ,...,x_{8} ]_{i} ) = [data_{1} ,...,data_{272847} ]_{i} , \\ i \in (0,900] \\ \end{gathered} $$

In Eq. (1), *F* represents the twin model of the relationship between the predictive variable and the node stress of the roadheader. The variable *x* comprises 8 factors that influence the mechanical performance of the roadheader. These factors include the vibration acceleration of the cutting arm, the displacement of the piston rod of the swinging hydraulic cylinder, the displacement of the piston rod of the lifting hydraulic cylinder, the pressure of the left swinging hydraulic cylinder, the pressure of the right swinging hydraulic cylinder, the pressure of the lifting hydraulic cylinder, the speed of the cutting head and the torque of the hydraulic motor. The *data* array represents the node stress of the boring machine, which consists of 278,747 elements, equivalent to the number of finite element mesh. The variable *i* indicates the sample sequence number of the dataset.

### Physical space

**Figure 4:**
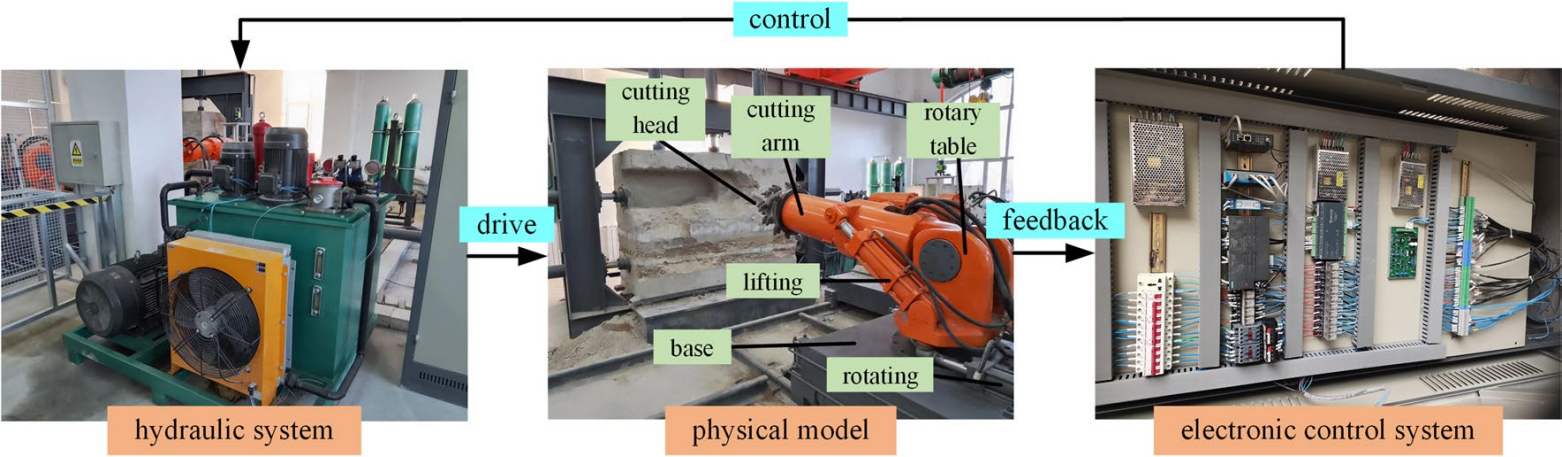
System of a boom-type roadheader.

The physical space of the roadheader is mainly composed of a hydraulic system, physical model, and electronic control system, as shown in Fig. 4. The roadheader utilized in the test contains a base, rotary table, cutting arm, cutting head, lifting hydraulic cylinder, and rotating hydraulic cylinder. The electrical control system outputs the control signal to the hydraulic system and drives the physical model of the roadheader to carry out the corresponding action. Sensor signals such as displacement, pressure, speed, torque, and acceleration are then acquired and fed back to the electronic control system. Subsequently, the electronic control system compares the feedback sensor data with pre-established safety thresholds. If the feedback sensor data fall within the acceptable range, the current cutting parameters will remain unaltered. However, if the feedback sensor data exceed the acceptable range, the swing speed of the cutting arm, cutting head speed, cutting pressure, and other parameters are reduced accordingly.

### Service space

#### Twin model construction

##### Method introduction

**Figure 5:**
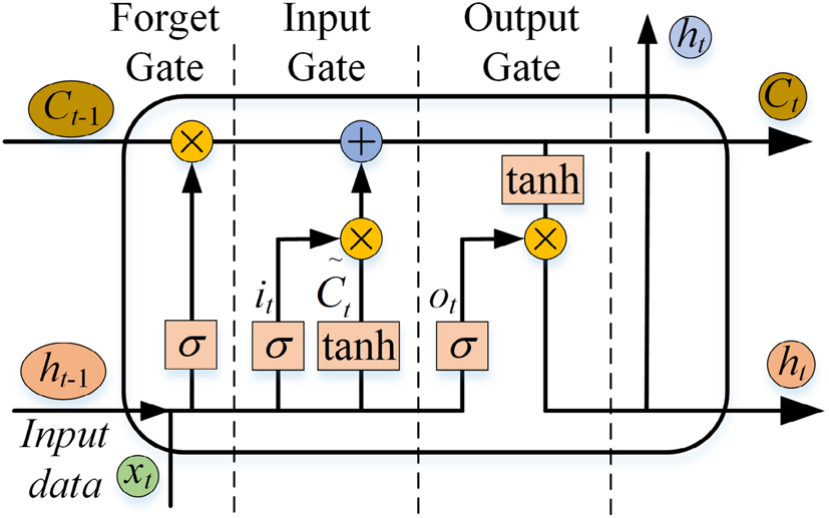
LSTM unit operation flow.

In the dynamic cutting process of a roadheader, the mechanical performance data are time dependent; that is, the output value at the current moment is related to the input value in the past period. The traditional feedforward neural network cannot handle this kind of timing information well. In contrast, the LSTM network^23^ can better control the transmission of information by introducing mechanisms such as forgetting gates, input gates, output gates and memory units; alleviate the problem of gradient disappearance in ordinary RNNs; has a stronger memory function; can process long sequence data; and has good generalizability. The operation flow chart of the LSTM unit is shown in Fig. 5.

As shown in Fig. 5, the forget gate determines whether the LSTM unit needs to forget some historical information. The formula is as follows:

2 $$ f_{t} = \sigma (W_{f} [x_{t} ,h_{t - 1} ] + b_{f} ). $$

In Eq. (2), *f*_*t*_ represents the information to be discarded, $\sigma$ represents the sigmoid function, *W*_*f*_ represents the forget gate weight, *b*_*f*_ represents the forget gate deviation,* x*_*t*_ represents the input at time *t*, and *h*_*t-*1_ represents the output at the previous time.

The input gate determines which signals flow into the LSTM unit at the current moment. The input gate generates input gate information *i*_*t*_ and $\tilde{C}_{t}$ according to the hidden layer state *h*_*t-*1_ at the previous moment and the input *x*_*t*_ at time *t*. The expression is as follows:

3 $$ \left\{ \begin{gathered} i_{t} = \sigma (W_{i} [x_{t} ,h_{t - 1} ] + b_{i} ) \hfill \\ \tilde{C}_{t} = \tanh (W_{C} [x_{t} ,h_{t - 1} ] + b_{C} ) \hfill \\ \end{gathered} \right.. $$

In Eq. (3), *W*_*i*_ and *W*_*C*_ represent the input gate weight, and *b*_*i*_ and *b*_*C*_ represent the input gate deviation. The output gate determines the output of the LSTM unit at the current time. The output gate combines the output values of the forget gate and the input gate to obtain the unit state *C*_*t*_ and hidden layer state *h*_*t*_ at time *t*, whose expressions are as follows:

4 $$ \left\{ \begin{gathered} C_{t} = f_{t} \cdot C_{t - 1} + i_{t} \cdot \tilde{C}_{t} \hfill \\ o_{t} = \sigma (W_{o} [x_{t} ,h_{t - 1} ] + b_{o} ) \hfill \\ h_{t} = o_{t} \cdot \tanh (C_{t} ) \hfill \\ \end{gathered} \right.. $$

In Eq. (4), *o*_*t*_ represents the output gate information, *W*_*o*_ represents the output gate weight, *b*_*o*_ represents the output gate deviation, and $\cdot$ represents element-by-element multiplication. Both the input and forget gates are controlled by the sigmoid function, and their output values are between 0 and 1, indicating the importance of a particular piece of information. The output gates are controlled by the sigmoid function and the tanh function.

LSTM networks have achieved great success in the field of timing information, and derivative versions of these networks have been proposed successively by scholars. In this paper, the bidirectional LSTM^24^ (Bi-LSTM) network is selected as the twin model. The Bi-LSTM network introduces the reverse layer and LSTM unit in the reverse direction. Learning the forward and backward information of time series data can result in obtaining sequence features more comprehensively and improving the modelling ability and robustness of the model.

##### Model training

**Figure 6:**
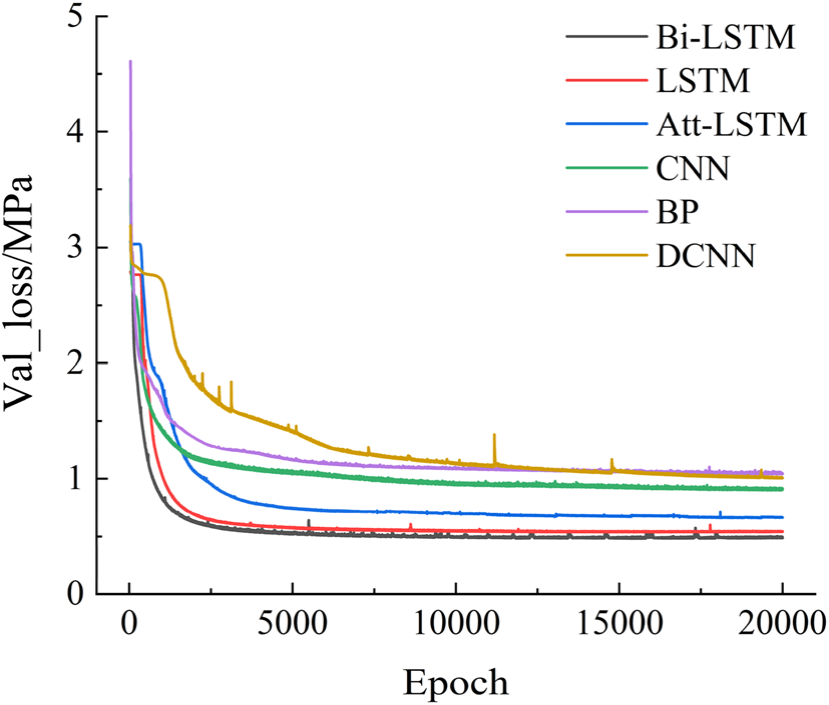
Comparison of the loss function curves of different models.

To prove the applicability of Bi-LSTM to the dataset of the mechanical performance of roadheaders, LSTM, self-attention mechanism LSTM (Att-LSTM)^25^, one-dimensional convolutional network (CNN)^26^, one-dimensional deconvolution network (DCNN)^27^ and feedforward neural network (BP)^28^ are selected as comparison models. Using the dataset generated by numerical simulation in Sect. "Dataset construction", the sample size is 900, and the ratio of the training dataset to the test dataset is set as 9:1. All the models are run in Intel-11700 k, NVIA-3080TI, and TensorFlow-GPU 2.4.0 environments with default hyperparameters and an average absolute error function as the loss function with the number of epochs set to 20,000. Figure 6 shows the change trend of the six models' loss functions on the test set.

As shown in Fig. 6, the performances of the BP network, CNN network and DCNN network on the test set are generally good. Since they have no explicit memory function, the memory function can be realized only by increasing the depth and width of the network and expanding the receptive field of the network to a certain extent. Compared with other networks, the three different types of LSTM networks converge at lower loss values, which proves that LSTM networks have certain advantages in processing time-series signals. The performance of the Att-LSTM network on the test set is worse than that of the LSTM network, mainly because after the addition of the attention mechanism, the Att-LSTM network has more trainable parameters than does the LSTM network and requires more samples and time to achieve the best performance. The Bi-LSTM network has the lowest loss on the test set and the fastest convergence speed. When training a dataset with a small number of samples, the Bi-LSTM network can capture the features in the timing information more comprehensively through forward and reverse learning so that the training model can converge faster and reach a lower loss value.

To evaluate the performances of the six models on the dataset of roadheader mechanical performance, the mean absolute percentage error (*MAE*), mean squared error (*MSE*), coefficient of determination (*R*^2^), and time used to predict a single sample are used to compare the prediction performances of the six methods. The *MAE* is used to measure the magnitude of the error between the predicted value and the actual value. Considering the direction of the error, the *MAE* is the average value of the absolute value of all the errors. For the fitting of the dataset of the mechanical performance of the roadheader, the *MAE* can represent the global fitting accuracy of the model. The *MSE* is the mean of the squared error of the predicted value and the actual value and can also be used to measure the global fitting accuracy of the model. However, the *MSE* is more sensitive to points with larger predicted error values, which magnifies the influence of some extreme error points on the overall error. *R*^2^ is an important index for determining the accuracy of model predictions. The closer the value is to 1, the more accurate the model prediction is.

In contrast, a lower *R*^2^ indicates a worse prediction result from the model. *R*^2^ can comprehensively reflect the prediction ability of the model. The mathematical expressions of the *MAE*, *MSE*, and *R*^2^ are as follows:

5 $$ MAE = \frac{1}{n}\sum\limits_{i = 1}^{n} {\left| {y_{i} - \hat{y}_{i} } \right|} , $$

6 $$ MSE = \frac{1}{n}\sum\limits_{i = 1}^{n} {\left( {y_{i} - \hat{y}_{i} } \right)^{2} } , $$

7 $$ R^{2} = 1 - \frac{{\sum\nolimits_{i = 1}^{n} {(y_{i} - \hat{y}_{i} )^{2} } }}{{\sum\nolimits_{i = 1}^{n} {(y_{i} - \overline{y}_{i} )^{2} } }}. $$

**Figure 7:**
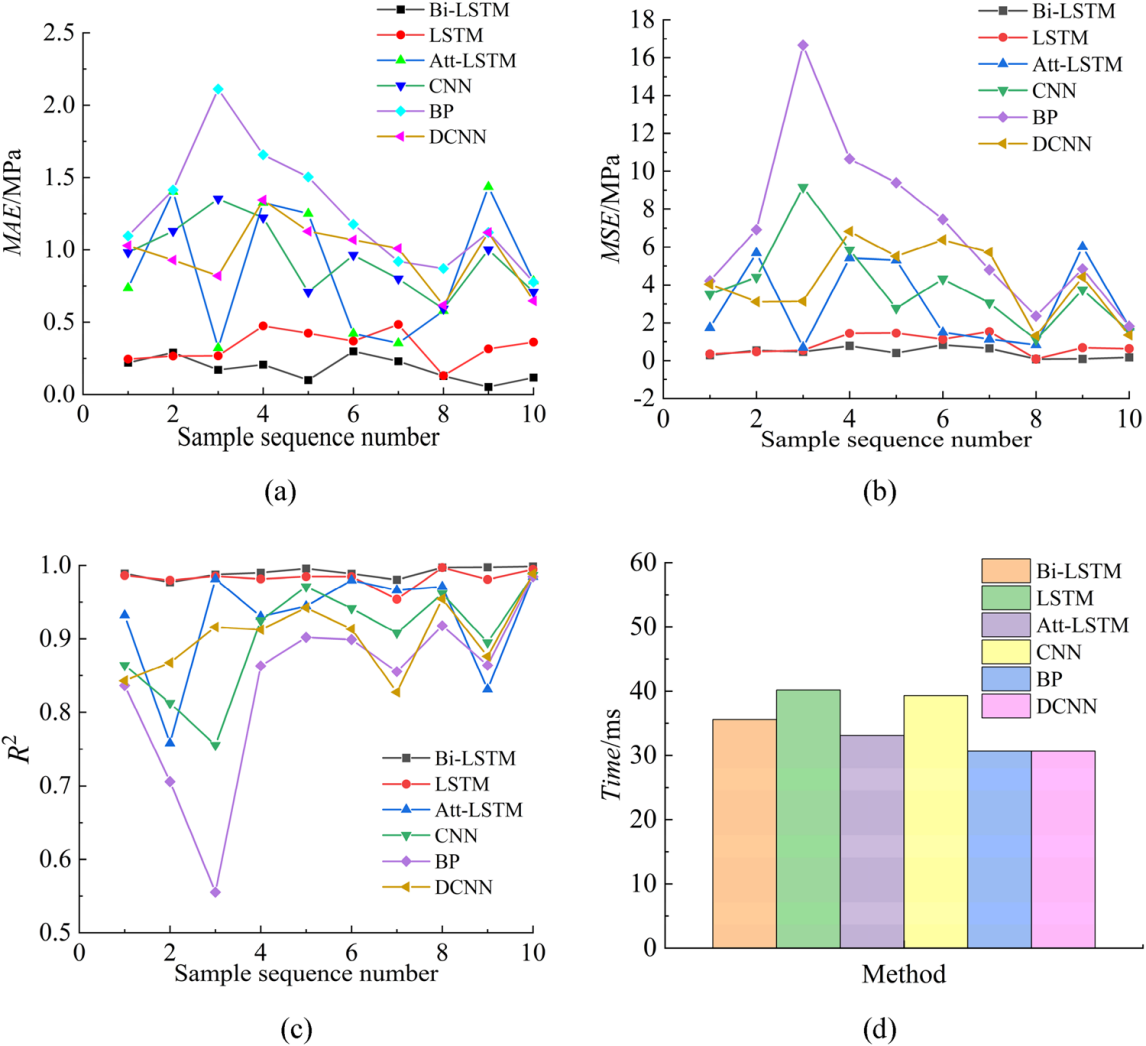
Index results of the six methods.

In the above equations, *y*_*i*_ is the actual value, $\hat{y}_{i}$ is the predicted value, $\overline{y}_{i}$ is the average of the actual values, and *n* is the number of samples. Ten groups of samples are randomly selected from the dataset to evaluate the *MAE*, *MSE* and *R*^2^. The times required by the six methods to predict a single sample are analysed. The test method involves inputting 1000 samples in a single cycle and testing the samples 10 times to determine the average. The evaluation indicators of the six methods are shown in Fig. 7.

As shown in Fig. 7, the DCNN, CNN, BP, and Att-LSTM networks exhibit poor performance in terms of the *MAE*, *MSE*, and *R*^2^, respectively, and all exhibit large fluctuations, indicating poor robustness and poor generalizability. Among them, the DCNN, CNN, and BP networks have poor index values at all sample points. It is proven that this approach is not applicable to a dataset on the mechanical performance of roadheaders. The performance of the Att-LSTM network on some sample points is the same as the optimal value. The Att-LSTM network has high feature extraction and fitting ability; however, it needs a large number of samples to obtain the best performance parameters, and overfitting occurs when training with a small sample dataset. According to the above figure, the LSTM network and Bi-LSTM network perform better in terms of all three evaluation metrics except time, but the LSTM network still has a certain degree of fluctuation, as indicated by its performance in terms of the *MAE*. Although the Bi-LSTM network also fluctuates in terms of the *MAE*, it benefits from the characteristics of bidirectional learning time series information. Its performance on the *MAE*, *MSE* and *R*^2^ evaluation indices is the best, and the fluctuation range is the smallest, which proves that it is more suitable for the mechanical performance dataset of roadheaders. The average values of the above four evaluation indicators are shown in Table 3.

**Table 3:** Mean value of the evaluation indices.

	DCNN	BP	CNN	Att-LSTM	LSTM	Bi-LSTM
*MAE*/MPa	0.9715	1.2648	0.9458	0.8614	0.3337	**0.1813**
*MSE*/MPa	4.1806	6.9121	3.9456	3.0099	0.8316	**0.4303**
*R*^2^	0.9042	0.8384	0.9022	0.9280	0.9829	**0.9902**
*Time*/ms	30.6796	**30.6764**	39.3179	33.1057	40.1745	35.5823

Significant values are in bold.

As shown in Table 3, the Bi-LSTM network has the best performance in terms of the *MAE*, *MSE* and *R*^2^. The BP network takes the least time to predict a single sample, and the Bi-LSTM network takes a longer time than the BP network. A single sample takes 5 ms longer, and the sampling interval of the roadheader sensor is 50 ms. Therefore, the time difference has little influence on the refresh frequency of the twin results. Based on the above results, the Bi-LSTM network is selected as the twin model of the "shape-performance-control" integrated digital twin system.

#### Control strategy

**Figure 8:**
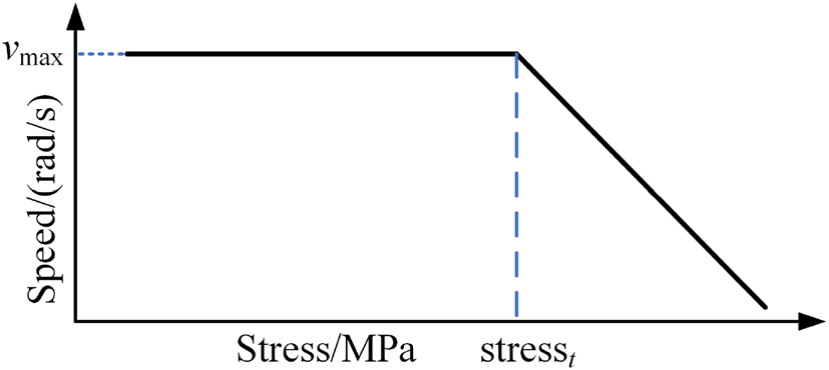
The stress corresponds to the speed of the cutting arm.

The load on the structural parts and the driving efficiency of the roadheader are strongly affected by the swing speed. To achieve safe cutting of the roadheader and improve driving efficiency, the swing speed of the cutting arm is adaptively controlled. In contrast to the existing adaptive cutting arm swing speed control method, the "shape-performance-control" integrated digital twin system of the roadheader feeds back the load state of the roadheader through the stress of the structural parts for the following reasons: Stress can comprehensively and intuitively reflect the load state of the roadheader. Vibration sensors, hydraulic cylinder pressure sensors, cutting motor current sensors, and other sensors can only be used to map the load state of the local area of the roadheader. Through continuous monitoring of the change in the stress in structural parts, possible failures or damage to the roadheader can be identified in advance to provide early warning. Thus, proper maintenance can be conducted, and the safety and reliability of the roadheader can be improved. The mechanical performance data of the roadheader are obtained through the twin model, the stress threshold is set, and the extreme stress value of the roadheader is converted into the corresponding cutting arm speed signal, that is, the movement speed of the rotating cylinder piston rod. The corresponding relationship is shown in Fig. 8.

**Figure 9:**
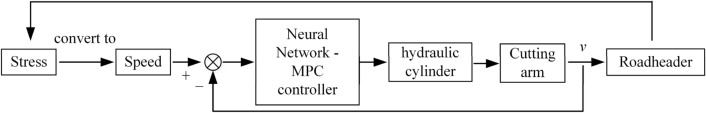
Swing speed control strategy for roadheaders.

When the stress is greater than the threshold value, the corresponding cutting arm speed decreases with increasing stress. When the stress is lower than the threshold value, the cutting arm speed is set to a constant value *v*_max_ to ensure cutting efficiency. In this paper, the stress threshold is set to 110 MPa, and the piston rod motion speed corresponding to the economic swing speed *v*_max_ is set to 5 mm/s. Taking the swing speed of the cutting arm as the control variable, a neural network MPC^29^ controller is used to control the servo valve, and the motion speed of the piston rod of the rotating hydraulic cylinder is subsequently controlled to dynamically adjust the swing speed of the cutting arm, as shown in Fig. 9.

**Figure 10:**
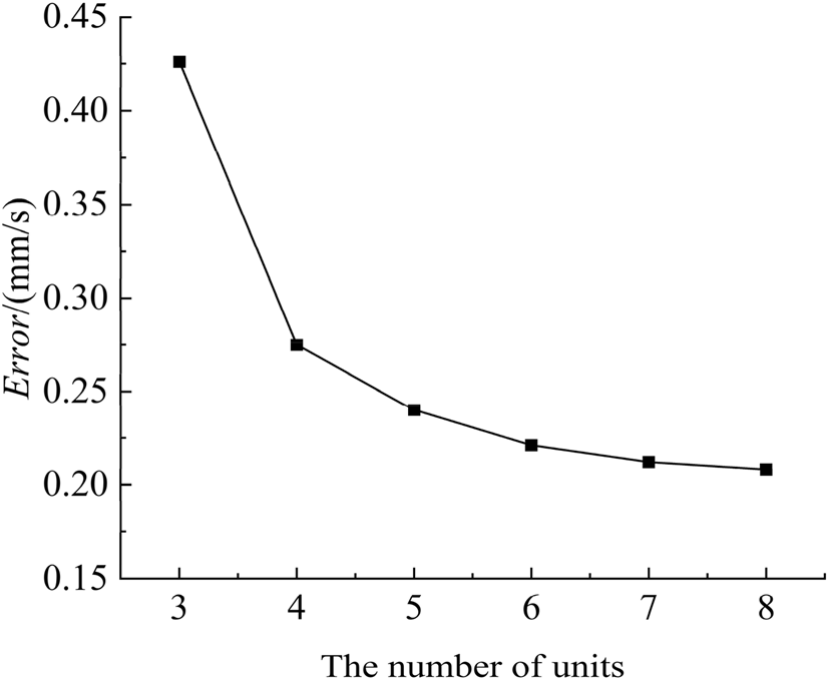
Relationship between the error and the number of hidden layer units.

In Fig. 9, the neural network-MPC controller is constructed in three steps:

1. A mathematical model relating the control quantity and the extension speed of the rotary cylinder piston is built. The dynamic swing speed control system of a roadheader is a complex, nonlinear and time-varying system. It is difficult to establish a mathematical model between servo valve signals (the servo valve signal here refers to the digital output signal of the PLC, which is used to control the analogue output of the servo valve (4–20 mA), and the digital output signal range of the Siemens PLC control servo valve is generally 5530–27,648) and piston rod speed by constructing the transfer function. Thus, the neural network is used to construct the mathematical model. First, the extreme stress values predicted by the twin model, the servo valve signal and the piston rod motion speed are collected. The piston rod motion speed and the maximum stress value predicted by the twin model are taken as the inputs of the neural network, and the output is the servo valve signal. The relationship between the control quantity and the piston extension speed is established by using the neural network. The parameters for the neural network have been determined through various experiments. On the premise that the fitting accuracy meets the requirements and the network is lightweight, the number of hidden layer is set to 1 layer. Figure 10 shown the relationship between the error and the number of hidden layer units. It is obvious that once the number of units exceeds 5, the error changes insignificantly. Therefore, the number of hidden layer units is set to 5.
2. The control objectives and constraints are determined. The expected piston rod movement speed is the control target, expressed as $v(t)$, and the actual piston rod movement speed is expressed as $v_{\varepsilon } (t)$. The goal of the control model is to obtain a $v_{\varepsilon } (t)$ that is as close as possible to $v(t)$ during the constant adjustment of the servo valve signal. In addition, there are several restrictions on the control system, such as the speed limit of the piston rod movement.
  8 $$ v_{\min } \le v(t) \le v_{\max } , $$

*v*_min_ and *v*_max_ represent the upper and lower limits of the piston rod speed, respectively. The limitations of the servo valve signal change rate can be expressed as follows:

9 $$ \Delta u_{\min } \le \Delta u(t) \le \Delta u_{\max } , $$

In Eq. (9), $\Delta u(t)_{\min }$ and $\Delta u(t)_{\max }$ represent the upper and lower limits, respectively, of the servo valve signal change rate, and $\Delta u(t)$ represents the servo valve signal change rate.

(3) The MPC is designed and solved. In rotating cylinder piston rod speed control, the MPC predicts the expected piston rod speed in the future according to the current state information and the expected piston rod speed. The optimal servo valve signal sequence is calculated using a numerical optimization algorithm to adjust the piston rod speed and ultimately adjust the cutting arm speed. The state equation of the rotating cylinder piston rod motion speed control system can be expressed as follows:
  10 $$ x(t + 1) = f(x(t),u(t)), $$

In Eq. (10), *x*(*t*) is the state variable of the system, *u*(*t*) represents the servo valve signal, and $f( \cdot , \cdot )$ represents the state transfer function, which indicates how the system state transitions to the next state under the action of the current state and control input. According to the determined control objectives and constraints, the optimization problem of the MPC can be expressed as follows:

11 $$ \left\{ \begin{gathered} \mathop {\min }\limits_{\Delta u(t)} (\sum\limits_{i = 0}^{{N_{p - 1} }} {\xi *(v(t + i|t) - r(t + i))^{2} } + \hfill \\ \sum\limits_{i = 0}^{{N_{c - 1} }} {\psi *\Delta u^{2} (t + i - 1)^{{}} )} \hfill \\ s.t.v_{\min } \le v(t + i|t) \le v_{\max } ,i \in [0,N_{p} - 1] \hfill \\ \Delta u_{\min } \le \Delta u(t + i - 1) \le \Delta u_{\max } ,i \in [1,N_{p} ] \hfill \\ \end{gathered} \right., $$

In Eq. (11), the optimization goal is to reduce the difference between the system output and the expected output as much as possible and, on this basis, reduce the control amplitude as much as possible. *N*_*p*_ represents the prediction time domain, *N*_*c*_ represents the control time domain, $\xi$ represents the output error weighting coefficient, $\psi$ represents the control amplitude weighting coefficient, $v(t + i|t)$ represents the piston rod movement speed at the prediction time *t* + *i*, *r*(*t* + *i*) represents the expected piston rod movement speed, and $\Delta u(t + i - 1)$ represents the change in the servo valve signal at the last time. The optimal control input parameters $\Delta u^{*} (t)$ are obtained via quadratic programming to realize high-precision control of the swing speed of the cutting arm of the roadheader.

### Twin space

**Figure 11:**
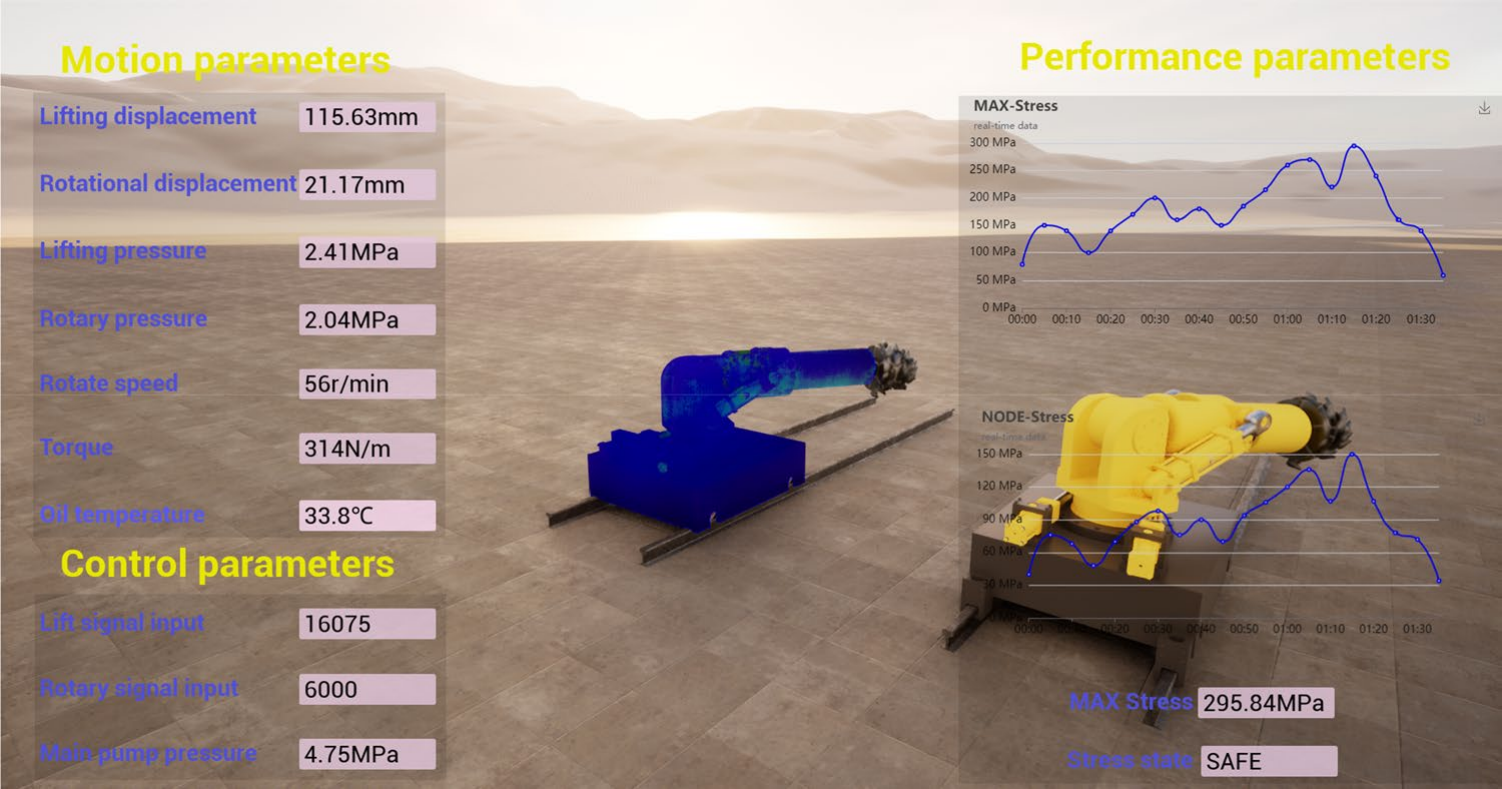
The "shape-performance-control" integrated digital twin platform of a boom-type roadheader.

With respect to the UE5 physical engine, an integrated digital twin platform of "shape-performance-control" is built. The objective of the twin space is to integrate the physical space and service space data and to determine the motion, performance and control parameters during the dynamic cutting process of the roadheader. What sets it apart from conventional dashboards is that a twin space constructed using the UE5 physics engine offers distinct advantages in 3D modeling, model modification, and model rendering. Its interface is shown in Fig. 11.

The twin platform comprises three submodules: a motion parameter module, a mechanical performance module, and a control parameter module. The motion parameter module can display cutting parameters such as the cutting head speed, torque, cylinder piston displacement, cylinder pressure, and oil temperature, which is convenient for observing the cutting state of a roadheader. The mechanical performance module can be used to visualize the stress information of the roadheader structural parts and display the information of all the node stresses, the selected node stress value curve, the extreme stress curve of the roadheader, and whether the stress value exceeds the yield strength of the material. The control module is responsible for displaying the speed information of the rotating cylinder and the input value of the servo valve signal.

In addition, the twin refresh time of the roadheader is evaluated, and the time of the single twinning cycle (multiple sensor data input—twin model operation—physics engine rendering) is 0.34 s, which satisfy the requirements of real-time monitoring of the mechanical performance of the roadheader.

## Test and results

To verify the feasibility of the "shape-performance-control" integrated twin system of roadheaders, a twin test system is built based on the laboratory of the Intelligent Industry and Mining Equipment.

**Figure 12:**
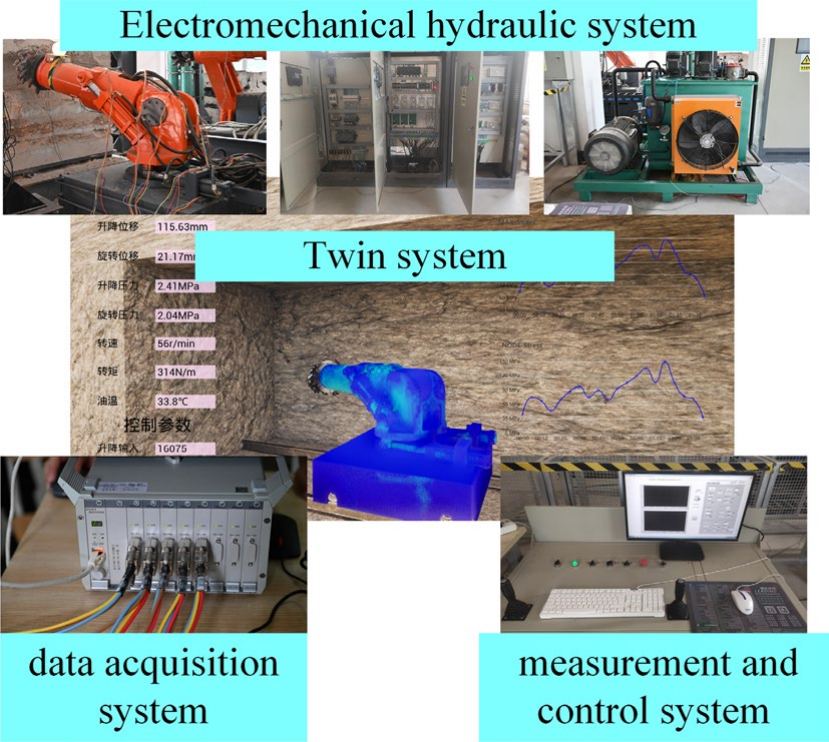
The twin test platform of the roadheader.

The twin test system of the roadheader includes an electromechanical, hydraulic system of the roadheader, a "shape-performance-control" integrated digital twin system of the roadheader, an upper computer measurement and control system, and a data acquisition system. The twin test system is shown in Fig. 12. The electromechanical and hydraulic system includes the physical model of the roadheader (cutting head, cutting arm, rotary table, hydraulic cylinder, base, etc.), the hydraulic system (servo valve, hydraulic oil, motor, variable pump, hydraulic pipeline, etc.), and the electronic control system. The measurement and control system of the upper computer is developed in LabVIEW. By establishing communication with the PLC at the operation desk, the control and monitoring of the electromechanical hydraulic system of the roadheader are realized. The data acquisition system includes vibration and strain information collection and saves the dynamic mechanical performance data of the roadheader during cutting. The "shape-performance-control" integrated twin system uses multi-sensor data to generate the twin of the roadheader. The swing speed control signal is input into the measurement and control system through the S7 protocol.

**Figure 13:**
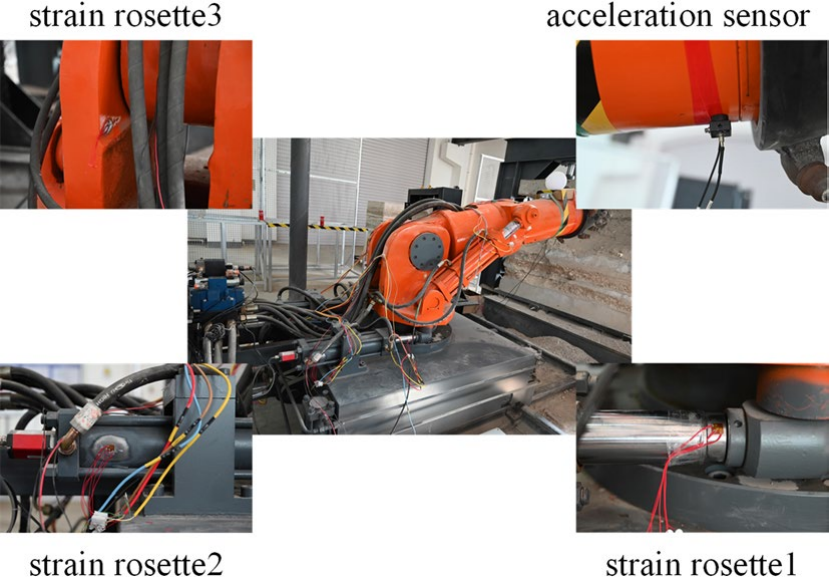
Strain and acceleration sensor layout.

The pressure, displacement, torque and speed sensors built in the roadheader are used to collect information via the measurement and control system of the upper computer. The vibration sensor and strain rosette need to be arranged in an additional step. The vibration sensor is placed near the cutting arm to collect the vibration acceleration signal of the cutting arm. The stress concentration position of the roadheader is determined according to the numerical simulation results, and the strain rosette is arranged such that the stress concentration is acceptable. The three positions of the roadheader rotary table, right rotation cylinder, and right rotation cylinder piston rod are selected for attaching the strain rosettes. The arrangement of the strain rosette and acceleration sensor is shown in Fig. 13.

### Accuracy verification and analysis of the twin model

**Figure 14:**
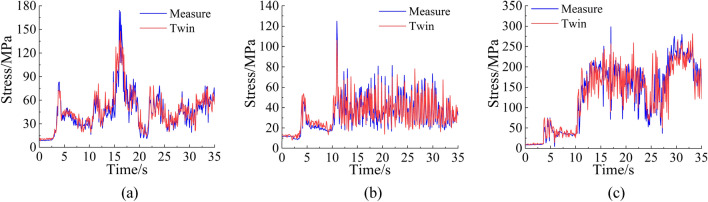
The test equivalent stress data are compared with the predicted equivalent stress data.

The sensor data collected during the test are input into the twin model to obtain the predicted data, and the predicted data corresponding to the strain rosette position are extracted. The equivalent stress can be calculated by using the strain rosette data and according to the generalized Hooke law and fourth strength theory. A comparison between the test equivalent stress data and the predicted equivalent stress data is shown in Fig. 14.

As shown in Fig. 14, the stresses in the piston rod, cylinder, and rotary table predicted by the twin model are basically consistent with the stress trend measured during the test. In the process of dynamic cutting, the twin model can still predict the stress state of each structural part well. The four indices, *MAE*, *R*^2^, mean absolute percentage error (*MAPE*), and root mean square error (*RMSE*), are used to quantify the difference between the predicted stress and measured stress. The specific quantitative evaluation indicators are shown in Table 4. The twin model is not accurate at predicting the performance of all structural parts during the cutting process. The deviation of the predicted stress value within 28–35 s of the rotary table is significant, and the overall stress variation trend is still consistent with the measured data. This is mainly because the twin model is obtained through the training of numerical simulation data. In the process of numerical simulation, to ensure the convergence and convenience of the results, the structure and material constitution of the three-dimensional model are often simplified, and the setting of the boundary conditions is idealized. However, these conditions are not completely consistent with a real scene, leading to differences between the numerical simulation results and actual situations. Moreover, whether the surface of the structural parts is clean and smooth, whether the strain rosettes are firmly pasted, the wire resistance, the channel drift, and other factors affect the authenticity of the stress data.

**Table 4:** Evaluation index statistics.

	*MAE*/MPa	*R*^2^	*MAPE*	*RMSE*/MPa
Piston rod	7.456	0.854	18.09%	9.512
Cylinder	4.722	0.888	15.47%	5.818
Rotary table	18.957	0.899	17.74%	25.357

Table 4 shows that the *MAE* and *RMSE* values for the cylinder and piston rod are low, indicating that the prediction error of the twin model at the positions of the cylinder and piston rod is relatively small. The *MAE* and *RMSE* of the rotary table are the largest, which is mainly due to the complex coupling and interaction between the rotary table and other parts of the roadheader. When the type of load alternates, the stress fluctuation range is large, and the change law is complex, so it is difficult to accurately predict by the twin model trained by numerical simulation only. The *R*^2^ and *MAPE* data of the three positions are consistent, indicating that the twin model has good generalizability and can adapt to the stress prediction of different structural parts.

### Control strategy verification and analysis

**Figure 15:**
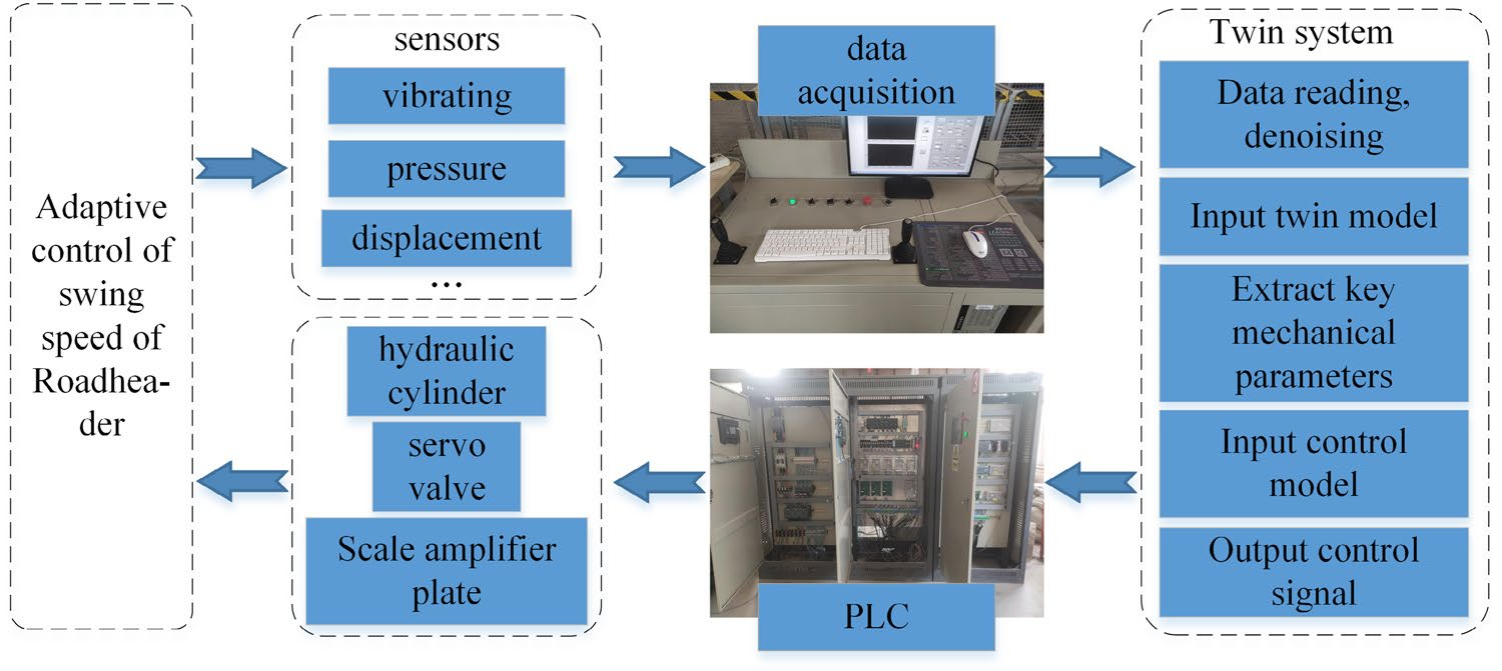
Swing speed adaptive control flow of the roadheader.

A data acquisition card is used to collect data from different types of sensors and transmit them to the upper-machine measurement and control system. The "shape-performance-control" integrated twin system of the roadheader transmits bidirectional data to the PLC in the upper computer measurement and control system based on the S7 protocol. Different sensing data types are input into the twin model to obtain the corresponding mechanical performance parameters. On this basis, the extreme stress value of the roadheader is extracted. Subsequently, the control model outputs the digital signal of the servo valve under the corresponding working conditions. The PLC in the upper computer measurement and control system receives the signal, converts it into a voltage signal, and amplifies it through the proportional magnifying board to promote the valve core of the servo valve, thereby changing the flow rate in the servo valve and realizing the purpose of controlling the extension and retraction speed of the rotating hydraulic cylinder piston rod. The swing speed adaptive control flow of the roadheader is shown in Fig. 15.

**Figure 16:**
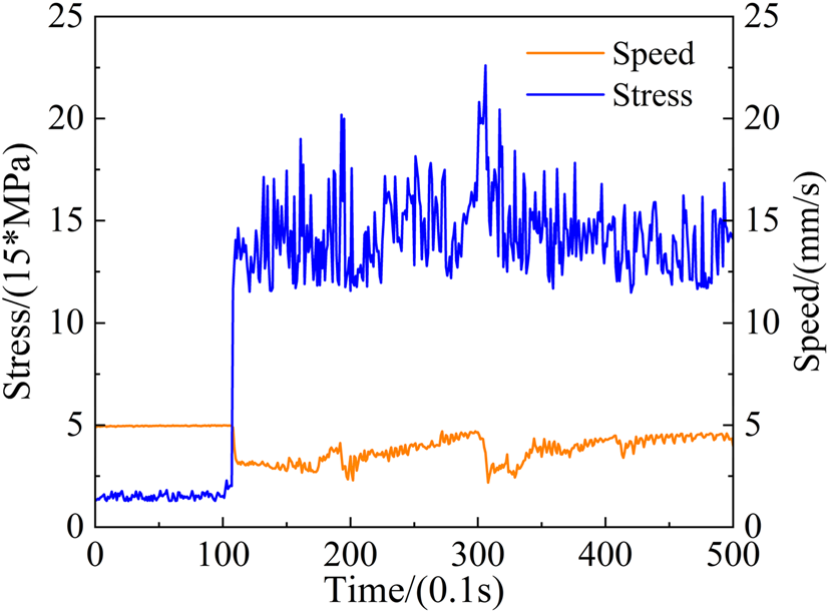
The result of adjusting the speed of the piston rod movement.

To validate the response speed of the proposed adaptive control strategy in response to changes in the structural stress and its efficacy in reducing peak stress in structural components, the roadheader twin test system is used to verify its control performance through horizontal cutting. The movement speed of the rotating cylinder piston rod and the maximum stress obtained by the twin model are recorded and analysed to create a corresponding curve, as depicted in Fig. 16.

As shown in Fig. 16, before 10 s, the roadheader is in a no-load state, and the movement speed of the piston rod, the swing speed of the cutting arm, is at the economical cutting speed. When the cutting head comes into contact with the coal and rock, the stress in the roadheader structural part increased rapidly, and the swinging speed of the cutting arm also decreased after approximately 0.3 s. Due to the temporal variability and anisotropy of coal and rock, the cutting resistance changes randomly, resulting in constant fluctuations in the extreme stress of the roadheader during the cutting process. Moreover, the swing speed of the cutting arm can still be adjusted accordingly, which verifies the feasibility of the adaptive control strategy for improving the swing speed of the roadheader.

## Discussion of the results

At present, the mechanical properties analysis of roadheaders or other mining machinery can be solved only by finite element or numerical analysis, which is not enough to meet the requirements of real-time decision-making. Therefore, an integrated digital twin system for the "shape-performance-control" of roadheaders is proposed. By inputting multi-sensor data into the twin model, real-time stress parameters of structural parts can be obtained. This process takes approximately 0.34 s, which effectively improves the computational efficiency of the mechanical properties of the roadheader and provides a way of thinking about real-time service performance monitoring of the roadheader. The experimental results show that the mean R^2^ of twin models is 0.88, and the mean absolute error between the twin data and the strain gauge data is 10.38 MPa. Moreover, the swing speed of the cutting arm can be controlled according to the extreme stress data predicted by the twin model. The test results reveal that the response speed of the control model is approximately 0.3 s. Furthermore, the damage to structural parts caused by overload can be avoided when the roadheader cuts under complex working conditions, providing a new basis for controlling adaptive cutting in roadheaders.

Predicting the mechanical performance of a roadheader is a complex task, and there are still several shortcomings in this study that need to be solved. For example, the prediction results of the twin model on the mechanical performance of some structural parts of the roadheader in the process of dynamic cutting are unsatisfactory, and the prediction error is sometimes significant. In future research, it is necessary to conduct customized research and apply various factors, such as different positions and loads; explore the correlation degree of different positions; and integrate this information into the model design and prediction process. Moreover, the twinning model should be modified according to limited and actual mechanical data to achieve more accurate and reliable twinning results. Although the proposed method still has room for improvement, it fills the gap in real-time monitoring of the mechanical performance of roadheaders. Additionally, a new adaptive cutting parameter control basis, which provides a way for intelligent roadheader monitoring, is proposed in this paper.

## Data Availability

The data that support the findings of this study are available on request from the corresponding author, upon reasonable request.

## References

[1] Wang, J. Development and prospect on fully mechanized mining in Chinese coal mines. *Int. J. Coal Sci. Technol.* **1**(3), 253–260 (2014). 10.1007/s40789-014-0017-2

[2] Zhiqiang, L. *et al.* Analysis of key technology and research path of full section boring machine for 1000 m vertical shaft with hard rock strata. *J. China Coal Soc.* **47**(08), 3163–3174 (2022).

[3] Hargrave, C. O., James, C. A. & Ralston, J. C. Infrastructure-based localisation of automated coal mining equipment. *Int. J. Coal Sci. Technol.* **4**(3), 252–261 (2017). 10.1007/s40789-017-0180-3

[4] Dolipski, M., Cheluszka, P. & Sobota, P. Investigating the simulated control of the rotateonal speed of roadheader cutting heads, relating to the reduction of energy consumption during the cutting process. *J. Min. Sci.* **51**(2), 298–308 (2015). 10.1134/S106273911502012X

[5] Hui, W., Zhen, W. & Di, W. A machine speed regulation system of the constant power based on RBF neural network PID control. *Meas. Control Technol.* **34**(11), 67–69 (2015).

[6] Kai, Z., Shishen, F., Miao, W. & Fulei, C. Simulation of control strategy for swing speed of roadheader’s cutting arm based on GA-BP network. *J. China Coal Soc.* **46**(S1), 511–519 (2021).

[7] Fei, T., Meng, Z., Yushan, L. & Nee, A. Y. C. Digital twin driven prognostics and health management for complex equipment. *Cirp-Annals* **2018**, 169–172 (2018).

[8] Grieves, M. Digital twin: Manufacturing excellence through virtual factory replication. *White Paper* **1**, 1–7 (2014).

[9] Xueguan, S. *et al.* Key technologies of shape-performance integrated digital twin for major equipment. *J. Mech. Eng.* **58**(10), 298–325 (2022). 10.3901/JME.2022.10.298

[10] Lai, X., Wang, S., Guo, Z., Zhang, C. & Song, X. Designing a shape-performance integrated digital twin based on multiple models and dynamic data: A boom crane example. *J. Mech. Des.* **143**(7), 1–15 (2021). 10.1115/1.4049861

[11] He, X. *et al.* M-LFM: A multi-level fusion modeling method for shape-performance integrated digital twin of complex structure. *Front. Mech. Eng.* **17**(4), 1–20 (2022). 10.1007/s11465-022-0708-0

[12] Latif, K., Sharafat, A. & Seo, J. Digital twin-driven framework for TBM performance prediction, visualization, and monitoring through machine learning. *Appl. Sci.* **13**(20), 11435 (2023). 10.3390/app132011435

[13] Xuhui, Z. *et al.* DT-driven memory cutting control method using VR instruction of boom-type roadheader. *J. China Coal Soc.* **2023**, 1–13 (2023).

[14] Xingwang, C., Yaoqiang, P., Jihua, Y. & Ruifeng, P. Research on digital twin technology of coal mine tunneling machine system. *J. Syst. Simul.* **2023**, 1–13 (2023).

[15] Jiajun, L. *Research and Implementation of TBM Virtual Tunneling System Based on Digital Twin* (Zhejiang University, 2021).

[16] Hua, D., Liangliang, Y., Zhaojian, Y. & Yiliang, W. Health prediction of shearers driven by digital twin and deep learning. *China Mech. Eng.* **31**(07), 815–823 (2020).

[17] Yan, W. *et al.* Construction of digital twin and parallel intelligent control method for excavation face. *J. China Coal Soc.* **47**(S1), 384–394 (2022).

[18] Songyong, L., Qiang, L., Yuming, C., Deyuan, M. & Qizhi, X. Design and research on mult-information monitoring system for roadheader. *J. China Coal Soc.* **48**(06), 2564–2578 (2023).

[19] Hongwei, M. *et al.* Key common technology of intelligent heading in coal mine roadway. *J. China Coal Soc.* **46**(01), 310–320 (2021).

[20] Wang, P. *et al.* Multiparameter control strategy and method for cutting arm of roadheader. *Shock Vib.*10.1155/2021/9918988 (2021). 10.1155/2021/9918988

[21] Wang, W., Yan, L., Wang, T., Guan, S., & Wang, D. Dynamic load identification method of rock roadheader using multi neural network and evidence theory. In *2016 IEEE International Conference on Mechatronics and Automation*, Harbin, China, 1238–1243 (2016).

[22] Dongjie, W. *et al.* Research on adaptive cutting control strategy of roadheader cutting arms. *China Mech. Eng.* **33**(20), 2492–2501 (2022).

[23] Hochreiter, S. & Schmidhuber, J. Long short-term memory. *Neural Comput.* **9**(8), 1735–1780 (1997). 9377276 10.1162/neco.1997.9.8.1735

[24] Tong, Y., Wu, P., He, J., Zhang, X. & Zhao, X. Bearing fault diagnosis by combining a deep residual shrinkage network and bidirectional LSTM. *Meas. Sci. Technol.* **33**(3), 034001 (2022). 10.1088/1361-6501/ac37eb

[25] Wang, Y., Huang, M., Zhu, X. & Zhao, L. Attention-based LSTM for aspect-level sentiment classification. *Proc. 2016 Conf. Empir. Methods Nat. Lang. Process.* **2016**, 606–615 (2016). 10.18653/v1/D16-1058

[26] Krizhevsky, A., Sutskever, I. & Hinton, G. Imagenet classification with deep convolutional neural networks. *Adv. Neural Inf. Process. Syst.*10.1145/3065386 (2012). 10.1145/3065386

[27] Noh, H., Hong, S., & Han, B. Learning deconvolution network for semantic segmentation. In *2015 IEEE International Conference on Computer Vision* (*ICCV*), 1520–1528 (2015).

[28] Rumelhart, D. E., Hinton, G. E. & Williams, R. J. Learning representations by back-propagating errors. *Nature* **323**, 533–536 (1986). 10.1038/323533a0

[29] Tao, S., Wei, X. & Daofei, L. CACC system based on MPC. *China Mech. Eng.* **28**(04), 486–491 (2017).

